# Insights into stress ulcer prophylaxis among non-critically ill patients: a tertiary hospital perspective from Sudan

**DOI:** 10.1186/s40780-025-00483-w

**Published:** 2025-08-11

**Authors:** Maram M. Elamin, Aisha Abdelraheem Mohamed, Moawia Elbalal Mohamed, Yousif B. Hamadalneel

**Affiliations:** 1 Department of Clinical Pharmacy and Pharmacy Practice, Faculty of Pharmacy, University of Gezira, Wad medani, Sudan; 2 Faculty of pharmacy, Wad Medani University for Science and Technology, Wad medani, Sudan; 3 Department of Internal Medicine, Faculty of Medicine, University of Gezira, Wad medani, Sudan

**Keywords:** Stress ulcer, Prophylaxis, Acid suppressive therapy, Non-critically, Sudan

## Abstract

**Background:**

Acid-suppressive therapy (AST) is often used without proper assessment of the need, dosage, or duration of treatment. This inappropriate use can lead to significant side effects, harmful drug interactions, and increased healthcare costs for patients.

**Objective:**

This study aimed to evaluate stress ulcer prophylaxis (SUP) among hospitalized adult patients who were non-critically ill.

**Method:**

This hospital-based, retrospective cross-sectional study was conducted in general internal medicine wards at Atbara Teaching Hospital, Sudan, from September to December 2024. The study included all hospitalized adult patients who received AST for SUP.

**Results:**

In total, 221 patients were evaluated in this study; among them, 136 (61%) were males and 75 (34%) were aged 61–80 years. Overall, only 84 patients (38%) were eligible for SUP, with pantoprazole 214 (97%) and intravenous (IV) route 215 (97.3%) being the most commonly used. Anticoagulant 48 (22%) and steroid or NSAID 20 (9%) use were the most and least the common risk factors based on ASHP criteria, respectively. A statistically significant association was found with polypharmacy (*P* < 0.001); 116 (52%) had polypharmacy, of whom 80 (69%) were eligible for SUP, and significant associations were also identified with other clinical characteristics, such as past medical history (*P* < 0.001) and sepsis (*P* = 0.013).

**Conclusion:**

This study revealed a high rate of inappropriate SUP prescriptions among non-ICU patients in a Sudanese hospital, with IV pantoprazole being the most used. These findings emphasize the need for evidence-based protocols, greater clinical pharmacist involvement, and interdisciplinary efforts to improve the appropriateness and cost-effectiveness of SUP use.

## Introduction

Stress ulcers are superficial inflammatory lesions that occur in the gastric mucosa when an individual experiences unusually high physiological stress [1]. In critically ill patients, the risk of significant bleeding from ulcers is estimated to range from 5 to 25%. Furthermore, 1–5% of stress ulcers can deepen enough to erode into the submucosa, resulting in clinically significant gastrointestinal bleeding, which may require a blood transfusion. In contrast, the risk of overt bleeding from stress ulcers in non-critically ill inpatients is less than 1% [2].

Disruption of the protective layer of the stomach can occur in critically ill patients through overproduction of uremic toxins, increased reflux of bile salts, compromised blood flow, and increased stomach acidity through gastrin stimulation of parietal cells [3]. Patients with serious illnesses are at high risk for gastric ulceration and bleeding; therefore, acid-suppressive therapy (AST), including proton pump inhibitors (PPIs) and histamine-2 receptor antagonists (H2RAs), is often used prophylactically to prevent gastric complications [4]. Guidelines recommend administering stress ulcer prophylaxis (SUP) to patients with critical illness who have additional risk factors for clinically significant stress-related gastrointestinal (GI) bleeding [5]. Several sources indicate that the majority of sick non-critically patients are given AST for SUP, whereas only a small proportion of high-risk patients may need it [6]. A study in Italy revealed that the main reasons for the overuse of AST are the unnecessary prevention of gastroduodenal ulcers in low-risk patients, SUP in non-critically, and incorrect diagnoses of acid-related disorders [7].

PPIs are often used without a proper assessment of the need, dosage, and duration of treatment [8]. They can cause significant side effects, lead to important drug interactions, and increase healthcare costs for patients [9]. This has become a crucial issue for regulatory authorities across various countries and continents. The problem is particularly pertinent considering the high costs incurred by both patients and governments [10], as well as the potential risks of iatrogenic harm resulting from adverse events or drug interactions in individuals who are taking multiple medications.

The continuous growth of the PPI market has raised significant concerns regarding the potential for inappropriate prescription of these medications. In Atbara, Sudan, no published study has addressed this issue. Therefore, this study aimed to conduct a comprehensive evaluation of SUP practices among hospitalized adult patients in the general wards of internal medicine at Atbara Teaching Hospital (ATH) in Sudan. By thoroughly assessing these practices, this study seeks to identify areas for improvement and develop evidence-based recommendations to optimize healthcare delivery concerning SUP in hospitalized patients.

## Method

### Study setting and duration

This hospital-based, retrospective cross-sectional study was conducted from September to December 2024 in the general internal medicine wards of ATH, located in River Nile State, Sudan. Established in 1908, ATH comprises nine wards within the Internal Medicine Department and has a total capacity of 210 beds. The hospital serves patients from various localities and rural areas across the River Nile State.

### Study participants

The study was conducted among hospitalized adult patients in general internal medicine wards who received AST for SUP. Patients were considered to have received AST for SUP if no documented GI indication was present and the AST was initiated presumptively as prophylaxis in the absence of active GI disease.

Patients diagnosed with stress ulcers, gastroesophageal reflux, or related disorders at the time of admission or with a prior diagnosis of these conditions were excluded from the study.

### Sample size and sampling technique

The study included all patients admitted to the hospital and receiving AST for SUP during the period from September to December 2024, with a total of 221 patients included.

### Data collection

Data were collected from patients and patients’ files during the hospitalization period via a specific data collection sheet. The data sheet consists of three sections. Section one includes patient demographics (age, sex, educational level, and the presence of health insurance) and clinical characteristics (diagnosis, past medical history, and length of hospitalization). Section two includes a medication review (past medications, current medications, and any medications that interfere with the gastrointestinal tract). Section three includes the risk factors, as illustrated by the American Society of Health-System Pharmacists (ASHP) guideline [11].

### Evaluation of SUP

The ASHP criteria were used to assess eligibility for SUP, and patients were considered eligible if they had at least one major risk factor or two or more minor risk factors [11]. The major and minor risk factors for SUP are detailed in Table 1.

**Table 1:** Major and minor risk factors for stress ulcer prophylaxis

Major risk factors	Minor risk factors
✓ Coagulopathy is defined as a platelet count lower than 50,000 or INR higher than 1.5 or a PTT higher than two times the control value ✓ Mechanical ventilation > 48 h ✓ Head trauma or spinal cord injury ✓ History of GI ulceration during the past year	✓ Sepsis ✓ Burn > 35% BSA ✓ Renal insufficiency ✓ Hepatic failure ✓ Use of anticoagulant ✓ History of Use of NSAIDS > 3 months ✓ Length of hospital stay (> 7 days) ✓ Glucocorticoid therapy (> 250 mg hydrocortisone or equivalent)

### Variable definition

Polypharmacy is the concurrent use of five or more medications regardless of route of administration [12]. In this study, polypharmacy included both newly prescribed and existing drugs, at the time of PPI initiation, excluding the AST itself.

### Statistical analysis

Data were analyzed via a statistical package for social science program software version 27 (SPSS Inc., Chicago, IL, USA). Frequencies (percentages) were used for categorical variables. After the applicability conditions were checked, the Pearson chi-square test and Fisher’s exact test, with a significance of 0.05, were used to identify factors associated with eligibility for SUP.

## Results

### Patients’ sociodemographic characteristics

Overall, 221 patients were evaluated in this study; among them, 136 (61%) were males, 75 (34%) were aged between 61 and 80 years, 66 (30%) had no formal education, and 133 (60%) lacked health insurance (Table 2).

**Table 2:** Patient sociodemographic and clinical characteristics

Characteristic	Frequency (*n* = 221)	Percentage %
**Age**		
18–40	61	28.0
41–60	71	32.0
61–80	75	34.0
> 81	14	6.0
**Education level**		
Uneducated	66	30.0
Primary	57	26.0
Secondary	65	29.0
Graduated	32	14.0
Post graduated	1	1.0
**Diagnosis**		
Infectious diseases	105	47.0
Renal diseases	26	12.0
Heart failure	23	10.0
Cerebrovascular accident	24	11.0
Liver Diseases	17	8.0
Anemia	9	4.0
Diabetes Mellitus	17	8.0
**Past Medical History**		
Free medical background	83	38.0
Hypertension	39	18.0
Diabetes mellitus	37	17.0
Heart diseases	23	10.0
Kidney diseases	19	9.0
Asthma	10	5.0
Liver diseases	9	4.0
Thyroid diseases	1	1.0

### Patient’s clinical characteristics

Overall, the majority of patients, 105 (47%), were diagnosed with infectious diseases, followed by 26 patients (12%) with renal diseases and 24 patients (11%) with cerebrovascular accident (CVA) diseases. With respect to past medical history, 83 patients (38%) had no prior history of chronic illness, 39 patients (18%) had a history of hypertension, and 37 patients (17%) had a history of type I or type II diabetes mellitus (Table 2). The majority of patients, 148 (67%), were hospitalized for less than seven days, whereas the remaining 73 patients (33%) had hospital stays exceeding seven days. In terms of medication usage, 57 patients (30%) used between 1 and 5 drugs, 133 patients (60%) used 6 to 10 drugs, and 31 patients (10%) used more than 10 medications.

### Eligibility for SUP and risk factors based on ASHP criteria

Overall, only 84 patients (38%) met the criteria for SUP indication, and 137 patients (62%) had taken AST without any evidence of indication. Pantoprazole was the most frequently used PPI for 214 patients (97%), followed by omeprazole for 6 patients (2%), and one patient (1%) received esomeprazole. Among those patients, 219 (99%) received a 40 mg dose, whereas 2 (1%) received a 20 mg dose. Twice daily doses were used in 131 patients (59%), followed by 88 patients (40%) who received once daily doses and 2 patients (1%) who received triple daily doses. The intravenous (IV) route was the most commonly used route and was administered to 215 patients (97.3%).

**Fig. 1:**
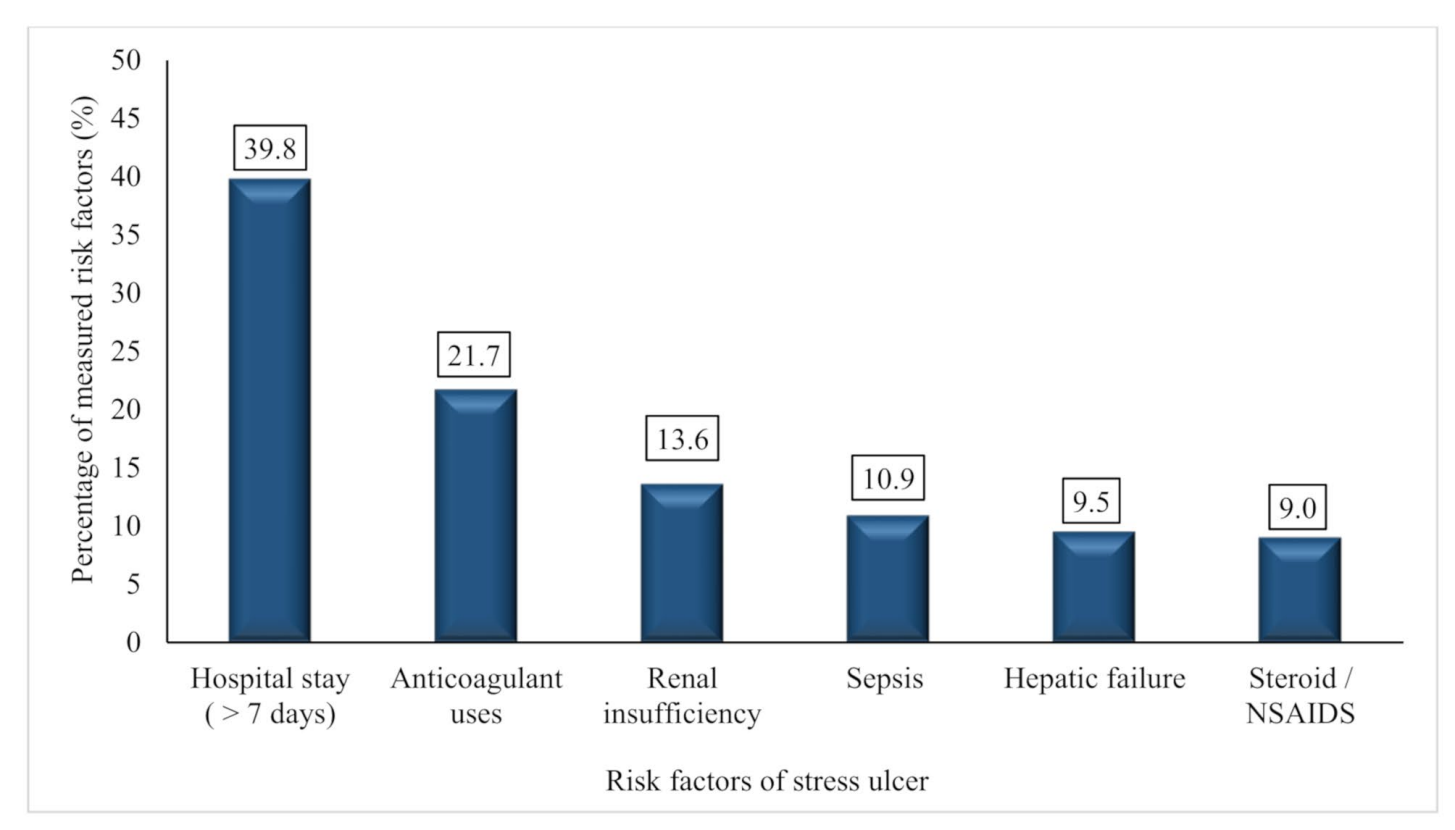
Distribution of ASHP-defined risk factors for stress ulcer prophylaxis among the studied patients (*N* = 221). ASHP: American Society of Health-System Pharmacists

Regarding the presence of risk factors, 79 patients (36%) had no identifiable risk factors, 70 patients (31%) had one risk factor, 57 patients (26%) had two, 13 patients (6%) had three, and 2 patients (1%) had four risk factors. Based on ASHP criteria, hospital stay for more than seven days and anticoagulant use were the most common primary risk factors, observed in 88 (39.8%) and 48 (21.7%) patients, respectively. In contrast, the least common risk factor associated with the use of hydrocortisone > 250 mg or equivalent and / or NSAIDS > 3 months was identified in 20 patients (9%) (Fig. 1).

### Factors associated with eligibility for SUP

The results revealed no statistically significant associations between SUP eligibility and patients’ sociodemographic characteristics (Table 3). However, significant associations were observed with several clinical characteristics. A statistically significant association was found with polypharmacy (*P* < 0.001); 116 patients (52.5%) had polypharmacy, of whom 80 (95.2%) were eligible for SUP. Significant associations were also identified with past medical history (*P* < 0.001) and sepsis (*P* = 0.013). In contrast, no significant association was found with hepatic failure (*P* = 0.643). Table 4 provides a detailed summary of the associations between SUP eligibility and patients’ clinical characteristics.

**Table 3:** Association between eligibility for stress ulcer prophylaxis and patient sociodemographic characteristics

Characteristic	Eligible *N* (%)	Not eligible *N* (%)	*P* value^*^
**Age**			0.168
18–40	16 (19.0)	45 (32.8)	
41–60	31 (36.9)	40 (29.2)	
61–80	31 (36.9)	44 (32.1)	
> 81	6 (7.1)	8 (5.8)	
**Gender**			0.776
Male	53 (63.1)	83 (60.6)	
Female	31 (36.9)	54 (39.4)	
**Education**			0.504
Uneducated	26 (31.0)	39 (28.5)	
Primary	25 (29.8)	32 (23.4)	
Secondary	20 (23.8)	46 (33.6)	
Graduated	13 (15.5)	19 (13.9)	
Postgraduate	0 (0.0)	1(0.7)	
**Health insurance**			0.571
Available	31(36.9)	57 (41.6)	
Not available	53 (63.1)	80 (58.4)	

Note: ^*^_,_ *p* value from chi-square and Fisher exact tests

**Table 4:** Association between eligibility for stress ulcer prophylaxis and patients’ clinical characteristics

Characteristic	Eligible *N* (%)	Not eligible *N* (%)	*P* value^*^
**Past medical history**			< 0.001
Free background	15 (17.9)	68 (49.6)	
Hypertension	19 (22.6)	20 (14.6)	
Diabetes	13 (15.5)	24 (17.5)	
Asthma	3 (3.6)	7 (5.1)	
Heart failure	13 (15.5)	10 (7.3)	
Liver disease	5 (6.0)	4 (2.9)	
Kidney disease	15 (17.6)	4 (2.9)	
Thyroid disease	1(1.2)	0 (0.0)	
**Length of hospital stay (> 7 days)**			< 0.001
≤ 7 days	31(36.9)	102 (74.5)	
> 7 days	53 (63.1)	35 (25.5)	
**Polypharmacy**			< 0.001
Yes	80 (95.2)	36 (26.3)	
No	4 (4.8)	101 (73.7)	
**Hydrocortisone > 250 mg or equivalent and / or NSAIDS > 3 months**			< 0.001
Yes	15 (75)	5 (3.6)	
No	69 (34.3)	132 (96.4)	
**Sepsis**			0.013
Yes	15 (17.9)	9 (6.6)	
No	69 (82.1)	128 (93.4)	
**Renal insufficiency**			< 0.001
Yes	24 (28.6)	6 (4.4)	
No	60 (71.4)	131 (95.6)	
**Hepatic failure**			0.643
Yes	9 (10.7)	12 (8.8)	
No	75 (89.3)	125 (91.2)	
**Anticoagulant use**			< 0.001
Yes	42 (50.0)	6 (4.4)	
No	42 (50.0)	131 (95.6)	

Note: ^*^_,_ *p* value from chi-square and Fisher exact tests

## Discussion

This hospital-based cross-sectional study evaluated the eligibility of SUP among adult non–ICU patients at a tertiary hospital in northern Sudan. Overall, only 38% of patients who received SUP in this study were eligible. This finding is greater than that of a retrospective study conducted among non-ICU patients at Soba Hospital, Khartoum, Sudan, where only 26% received SUP appropriately [13], and a cross-sectional study in Pakistan reporting 31.02% appropriate use of SUP [14], suggesting marginally improved compliance with SUP guidelines in our hospital setting. However, the results are comparable to those of a cross-sectional study conducted in the medical wards of Gondar Hospital, Ethiopia, which reported that 36.6% of SUP cases were appropriate [15], highlighting comparable challenges in non-ICU patients within similar healthcare systems. In contrast, higher rates of appropriate SUP use were reported in a cross-sectional study in the orthopedics department in northwestern China 66.9% [16], a retrospective cross-sectional study in a tertiary hospital in Saudi Arabia (54%) [17], a cross-sectional study at Dessie Referral Hospital in Northeast Ethiopia (50%) [18], and among surgical patients at Debre Berhan Hospital, Ethiopia (44%) [19], highlighting the necessity for better execution of guideline-based medication prescriptions. To address this problem, institutional guidelines and training on the appropriate indications for SUP usage need to be strengthened. Clinical pharmacists play a crucial role in reviewing prescriptions and guiding appropriate SUP use, as evidenced by a systematic review and a quasi-experimental study from Egypt, both of which demonstrated that pharmacist-led interventions significantly reduced inappropriate or non adherent SUP prescriptions, lowered hospital costs, and did not increase adverse effects [20, 21]. Additionally, incorporating clinical decision support tools and providing regular training for healthcare professionals may greatly increase compliance with SUP guidelines, minimize unnecessary medication usage, and avert possible adverse effects or increase healthcare expenses. 

This study revealed that a hospital stay of more than seven days and anticoagulant use were the most common primary risk factors for stress ulcers among the studied patients based on the ASHP criteria. This finding contrasts with other cross-sectional studies conducted among non-ICU patients, where different minor risk factors were more prevalent, such as NSAID or high-dose aspirin use in Sudan [13], renal insufficiency in Iran [3], and NSAID use in Gondar, Ethiopia [15]. This variation might indicate disparities in prescribing habits, patient demographics, or underlying health conditions across different areas. Recognizing key risk factors such as a long hospital stay and a significant rate of anticoagulant usage in this research is essential for customizing SUP, emphasizing the need for focused risk evaluations and ongoing prescription assessments to guarantee suitable usage, particularly with the support of clinical pharmacists. 

In this study, polypharmacy was significantly associated with SUP eligibility. It was observed in 52.5% of the patients. This finding supports findings from a study conducted at a German university hospital, which linked gastrointestinal bleeding to polypharmacy [22], and a study in France that associated PPIs with multiple morbidities in older patients [23]. Additionally, a cross-sectional study conducted in medical wards in Gondar, Ethiopia, identified central nervous system disorders and length of hospital stay as factors associated with the appropriateness of SUP use [15]. These findings underscore the importance of considering polypharmacy and comorbidities in SUP assessment, with thorough medication reviews, particularly by clinical pharmacists, enhancing appropriate prescribing and reducing unnecessary use [24]. 

In this study, all patients received PPIs as SUPs, with pantoprazole being the most commonly used. This finding is consistent with a retrospective cross-sectional study conducted in the Department of Internal Medicine at Soba Hospital, Khartoum, Sudan, and a cross-sectional study in a non-ICU tertiary hospital in Saudi Arabia, both of which reported that all patients received PPIs for SUP [13, 17]. Similarly, a cross-sectional study in Gondar, Ethiopia, reported that 92.7% of patients received PPIs, whereas 7.3% were given H2RAs [15]. In contrast, a study among surgical patients at Debre Berhan Hospital, Ethiopia, reported that 50% of patients received H2RAs [19]. However, the absence of H2RAs in the Sudanese market restricts treatment choices, resulting in a possible excessive dependence on PPIs, which, while beneficial, could introduce unnecessary risks and expenses, underscoring the importance of local guidelines and the participation of clinical pharmacists in encouraging suitable, safe, and economical SUP use. In the present study, the IV dosage form was used for nearly all patients. This finding aligns with a cross-sectional study conducted at Debre Berhan Hospital, Ethiopia, where the IV route was also the most commonly used route [19]. Similarly, a cross-sectional study from Dessie Referral Hospital in Northeast Ethiopia reported that 90% of patients received SUP via the IV route [18]. However, the 2020 guidelines from the American College of Clinical Pharmacy state that oral PPIs are as effective as IV PPIs in maintaining equivalent pH levels [11]. Furthermore, a randomized controlled trial confirmed the equal efficacy of oral and IV PPIs and recommended oral administration to reduce healthcare costs [25]. These results indicate the need to reevaluate standard IV administration for SUP in non-critically ill patients, encouraging the use of oral PPIs when possible to increase resource use and decrease avoidable healthcare costs.

This research emphasizes the pressing necessity of enhancing SUP prescription methods for non-ICU patients in Sudanese hospitals, where excessive use of AST, especially PPIs, indicates a discrepancy between clinical practices and recognized guidelines. Creating and applying clear, evidence-based local SUP regulations, along with decision-support tools, is vital for directing suitable prescribing. The incorporation of clinical pharmacists as essential team members can significantly assist in the evaluation of prescriptions, the performance of medication reconciliation, and the training of healthcare providers, which ultimately minimizes improper SUP usage, improves patient safety, and maximizes healthcare resources. Furthermore, promoting the use of oral instead of intravenous PPIs when appropriate can decrease expenses and mitigate unnecessary risks, whereas teamwork among various disciplines is essential for achieving enduring enhancements.

This research emphasizes the necessity for additional studies to inform enhancements in SUP practices within Sudan. Future research should engage various hospitals to evaluate national prescribing trends and aid in creating standardized guidelines. Interventional studies, such as pharmacist-driven programs and decision-support systems, are crucial for improving suitable SUP utilization. Moreover, analyses of cost-effectiveness, studies focused on patient-centered outcomes, and research into obstacles to following guidelines are needed. Future studies need to investigate the clinical effects of improper acid-suppressive therapy to guarantee safer and more effective patient treatment.

### Strengths and limitations

The research utilized a full enumeration sampling approach, incorporating all eligible patients during the study timeframe, thereby improving the representativeness of the results. Moreover, applying recognized international standards (ASHP criteria) for assessing SUP suitability provides reliability and uniformity to the findings.

However, this research has a number of limitations. As a single-center, cross-sectional study, the results may not apply to other hospitals or healthcare environments throughout Sudan. The observational design restricts the capacity to determine causal links between SUP usage and clinical results. Furthermore, the research did not evaluate long-term results or possible adverse effects of inappropriate AST, nor did it incorporate qualitative information regarding healthcare providers’ prescribing practices or understanding. The lack of alternative treatments, such as H2RA, in the local market might have impacted prescribing practices, potentially restricting the relevance of the findings to environments with greater medication availability. Additionally, the logistic regression model failed to converge, likely due to perfect prediction (e.g., almost all patients with polypharmacy are “eligible”) and a low event-per-variable ratio, resulting in inflated coefficients. Therefore, the ‘Variables in the Equation’ table is not valid for interpretation.

## Conclusion

This research demonstrated a significant occurrence of inappropriate SUP prescriptions among non-ICU patients in a tertiary hospital in Sudan, with most patients receiving AST lacking clear clinical justifications. Pantoprazole is the most frequently utilized medication and is given mainly intravenously, leading to avoidable healthcare expenses and possible risks for patients. The results highlight the critical necessity of establishing evidence-based local protocols, strengthening the involvement of clinical pharmacists in healthcare teams, and encouraging the judicious use of SUP to increase patient safety and maximize resource use. Addressing these challenges via interdisciplinary cooperation, ongoing training, and clinical decision-support systems is crucial for attaining more suitable and economical SUP practices.

## Data Availability

The datasets used and/or analyzed during the current study are available from the corresponding author upon reasonable request.
